# Enhancing Tensile Performance of Lithium Slag Geopolymers Using Hybrid Fibers and Modified Multi-Walled Carbon Nanotubes

**DOI:** 10.3390/ma19010213

**Published:** 2026-01-05

**Authors:** Qing Li, Chong Deng, Yali Hu, Mingxing Luo, Daopei Zhu, Cai Wu

**Affiliations:** 1 School of Civil Engineering, Hubei Engineering University, Xiaogan 432000, China; liqing@hbeu.edu.cn (Q.L.); whanst@whut.edu.cn (M.L.); 2 School of Software Engineering, Jiangxi University of Science and Technology, Nanchang 330013, China; m13667055100@163.com (C.D.); daopei.zhu@jxust.edu.cn (D.Z.); 3 School of Materials Science and Engineering, Chongqing University, Chongqing 400044, China; 18051488237@163.com

**Keywords:** Lithium slag geopolymer, hybrid fibers, lithium mica mine smelting tailings, modified multi-walled carbon nanotubes

## Abstract

This study investigates the synergistic effects of hybrid fibers and functionalized multi-walled carbon nanotubes (MWCNTs) on the mechanical and microstructural properties of lithium slag–based geopolymers (FL-EGC). Unlike conventional studies that focus on single reinforcement strategies, this work combines nanoscale modification with macroscale fiber reinforcement to overcome the inherent brittleness of geopolymers. Results show that while hybrid fibers and MWCNTs reduce flowability, the incorporation of 2.5% PVA, 1.0% steel fibers, and 0.15% MWCNTs yielded the best balance of performance, improving ultimate tensile stress by 12.7%, strain by 69.2%, and specific fracture energy by 78.2%. Microstructural analysis confirmed that MWCNTs enhanced crack-bridging and matrix densification, while hybrid fibers improved strength and ductility. These findings demonstrate a novel reinforcement pathway for developing sustainable, high-performance geopolymers from industrial by-products, providing both theoretical insights and practical guidance for green construction materials.

## 1. Introduction

Lithium slag, a high-alumina industrial by-product generated during lithium extraction [1], and fly ash are increasingly recognized as promising aluminosilicate precursors for geopolymer binders [2]. Geopolymers synthesized from such industrial wastes exhibit a three-dimensional aluminosilicate network and offer significant advantages over ordinary Portland cement, including reduced energy consumption and environmental impact. Consequently, lithium slag–based geopolymers have attracted growing attention as sustainable alternatives for cementitious materials in construction applications [3,4,5].

Compared to ordinary silicate cement (OPC), geopolymer is affected by high brittleness, which prevents its engineering application from being maximized economically [6]. Therefore, researchers proposed a fiber-reinforced geopolymer composite (EGC) [7,8,9,10,11,12,13,14] that combines the excellent properties of engineered cement composites (ECCs), which strengthen geopolymer composites through the interaction of fibers with the geopolymer matrix [15,16,17], effectively solving the high brittleness and enhancing the crack control ability. Fibers such as polypropylene fibers (PP), polyvinyl alcohol fibers (PVA), and steel fibers can be used as fiber reinforcing materials, e.g., Nuaklong et al. [18] used steel fibers as reinforcement materials to strengthen geopolymer concrete; in addition, a part of the researchers proved that different types of fibers can also strengthen geopolymers [19,20,21,22,23,24,25,26,27]. Based on the design concept of ECC [28], combining it with the excellent properties of geopolymers, it is due to the reinforcing effect of fibers that the brittleness of geopolymers is suppressed. Therefore, a large number of researchers have reported the performance of EGC using fibers with different moduli, which is important for the study of the advancement of green energy and energy-efficient building materials.

However, some researchers have found that EGCs prepared using fibers with different elastic moduli and lengths behave differently, with EGCs with low elastic modulus fibers exhibiting high ductility, low tensile strength, and the formation of multiple microcracks during the damage process [21,22], while EGCs with high elastic modulus exhibit high tensile strength, low elongation, and fewer cracking patterns [29,30]. Therefore, some researchers have incorporated different types of fiber composites to make EGCs capable of both high tensile strength and high ductility [29]. Zhang et al. [31] prepared fiber-reinforced geopolymers using four types of fibers (steel, PVA, PP, and regenerated polypropylene (RPP)), and the results measured in the study showed that steel and RPP fibers had the best reinforcing effect. Khan et al. [32] prepared fiber-reinforced EGCs using different contents of steel and high-strength polyethylene (HSPE) fibers and found that the best reinforcement was achieved when the steel fiber content was 1.60%, and HSPE content was 0.40%. Therefore, this indicates that the incorporation of different ratios of fibers into EGC can effectively improve the mechanical properties of the material.

Meanwhile, nanomaterials used for the modification of reinforced concrete have also been rapidly developed in recent years, and researchers have found that nanomaterials such as silica nanoparticles, carbon nanotubes (CNTs), graphene nanosheets, and so on, can be used as modified reinforcement for concrete and EGC [33,34,35,36,37,38,39,40,41,42,43,44]. The multi-walled carbon nanotubes (MWCNTs) obtained by modifying carbon nanotubes have excellent mechanical properties, which have been applied in EGC by some researchers to effectively improve the mechanical properties and microstructure of geopolymers [45,46]. For example, Khater et al. [47] observed that the addition of 0.1% MWCNTs increased the tensile and flexural properties and strength of the geopolymer and significantly reduced the drying shrinkage and water absorption. However, after continuing to increase the MWCNTs content, the MWCNTs began to aggregate in the matrix, negatively affecting the structure of the formed geopolymer. Also, some researchers have investigated the incorporation of both fibers and nanomaterials into the geopolymer, and it was found that the synergistic preparation of MWCNTs with fibers also strengthens the EGCs; e.g., Li et al. [48] found that the EGCs prepared with 0.1 wt.% of MWCNTs had higher flexural and compressive strengths. Due to the presence of MWCNTs, the carbon nanotubes only undergo bending deformation in bending or even when the external environment is damaged, so MWCNTs can enhance the ductility of composites from a microscopic point of view. Meanwhile, the nanomaterials themselves have a strong nano-nucleation effect and nanofilling effect, which can greatly enhance the compressive properties of EGCs. However, the reinforcement mechanism of geopolymer synergistic hybrid fibers and modified materials is extremely complex, and at the same time, it has to comply with the design of high-performance and green energy saving, and this kind of research has limited reports so far.

Therefore, based on the above effects, the tensile properties of geopolymers with fly ash microspheres and lithium slag will be investigated in this study using different volume ratios of hybrid fibers (steel fibers, PVA fibers) and different mass fractions of MWCNTs. The main objectives of the study were to rationalize the secondary use of industrial wastes containing lithium slag (LS) and to produce green FL-EGC to meet the requirements, to enhance the tensile strength and crack control of FL-EGC geopolymers, to differentiate them from conventional, high tensile strength geopolymers, and to require research and analysis of the enhancement mechanism at the microscopic level. The study tested the flowability of FL-EGC fresh slurry. The tensile properties of FL-EGC were analyzed and studied using uniaxial tensile tests with initial cracking stress–strain behavior up to failure and strain-specific fracture energy. Cracking characteristics were also analyzed, which can better corroborate the mechanism of enhanced crack control of FL-EGC by MWCTNs and hybrid fibers. Finally, the microscopic morphology of FL-EGC was analyzed using the FESEM (Zeiss Sigma, Panako, UK) experimental study, and the potential mechanism of tensile properties of geopolymer materials with hybrid fibers was analyzed from the micro-interface.

Based on the literature, previous studies have demonstrated that (i) single-type fiber reinforcement can improve either tensile strength or ductility of geopolymer composites [45], but often at the expense of the other; (ii) nanomaterials such as MWCNTs can enhance matrix densification and microcrack resistance [46], yet their effectiveness is highly sensitive to dosage and dispersion; and (iii) although several studies have explored the combined use of fibers and nanomaterials, most of them focus on conventional geopolymer systems (e.g., fly ash or metakaolin–based matrices) [47] and primarily report compressive or flexural behavior [48]. However, systematic investigations into the tensile behavior and crack control mechanisms of lithium slag–based geopolymer composites incorporating both hybrid fibers (with different elastic moduli) and modified MWCNTs remain very limited. In particular, the synergistic reinforcement mechanism across multiple scales—from nanoscale crack bridging and matrix densification to macroscale fiber pull-out and strain-hardening behavior—has not been fully clarified for lithium slag–based systems. To address this gap, the present study focuses on lithium slag–based engineered geopolymer composites reinforced with steel fibers, PVA fibers, and functionalized MWCNTs. By combining uniaxial tensile testing, crack pattern analysis, and microstructural characterization, this work aims to (1) optimize the hybrid reinforcement design for enhanced tensile strength and ductility, and (2) elucidate the multi-scale synergistic toughening mechanisms in sustainable geopolymer composites.

## 2. Materials and Experimental Methods

### 2.1. Raw Materials

#### 2.1.1. Lithium Slag and Four Cementitious Materials

**Figure 1:**
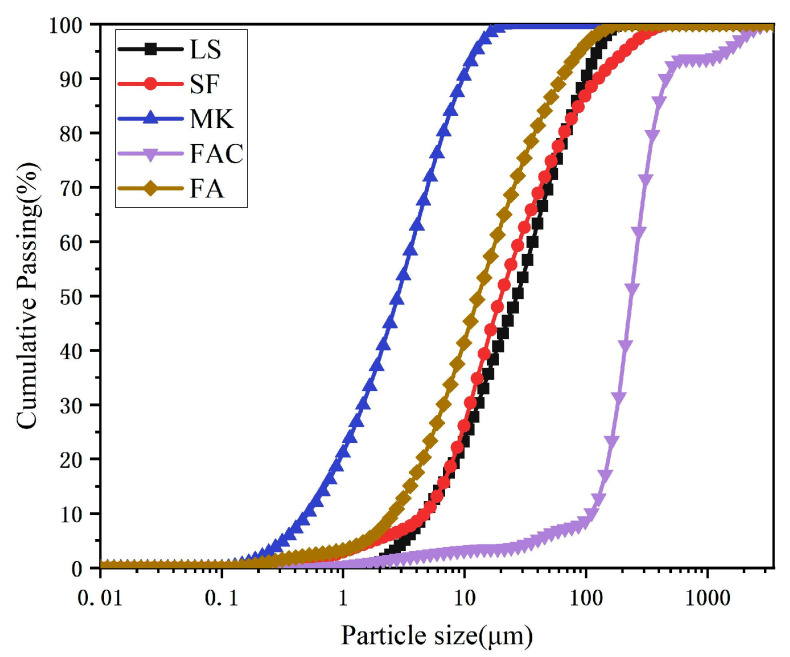
Particle size analysis of each material.

**Figure 2:**
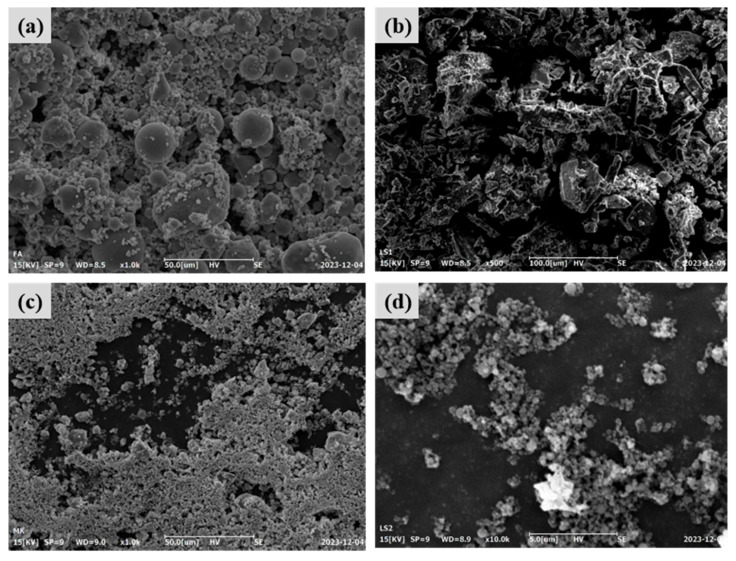
Microscopic images of the four feedstocks. (**a**) FA, (**b**) LS, (**c**) MK, (**d**) SF.

The lithium slag (LS) used in this study was obtained from a mine ore in Yichun, Jiangxi Province, which is a waste slag from smelting lithium mica tailings. In Table 1, the chemical composition analysis of the LS and the four cementitious materials used in this study is shown. The four cementitious materials include fly ash (FA), fly ash microsphere (FAC), metakaolin (MK), and silica fume (SF). The high SiO_2_ and Al_2_O_3_ content in LS, FA, FAC, MK, and SF is favorable for geopolymerization, providing sufficient reactive silica and alumina for polycondensation. FA has an average particle density of 2100 kg/m^3^ and a particle size range of 10–100 μm, and FAC, as the main lightweight filler of FL-EGC, has an average particle density and crushing strength of 550 kg/m^3^ and 15.0 MPa, and a particle size range of 10–300 μm. The measured density of the prepared FL-EGC in this study was below 1950 kg/m^3^, which meets the definition of lightweight aggregate concrete according to the Chinese standard JGJ/T 12-2019 [24]. Both MK and SF were in powder form with high specific surface area and amorphous silica content, which are indicative of high reactivity. As shown in Figure 1, the particle size distributions of the five raw materials were measured by a laser particle sizer after the dispersion treatment. Meanwhile, FA, LS, MK, and SF were observed by FESEM, as shown in Figure 2; FA was spherical, LS was irregularly lumpy, MK was lamellar, and SF was nearly spherical.

#### 2.1.2. MWCNTs, PVA Fibers, and Steel Fibers

**Figure 3:**
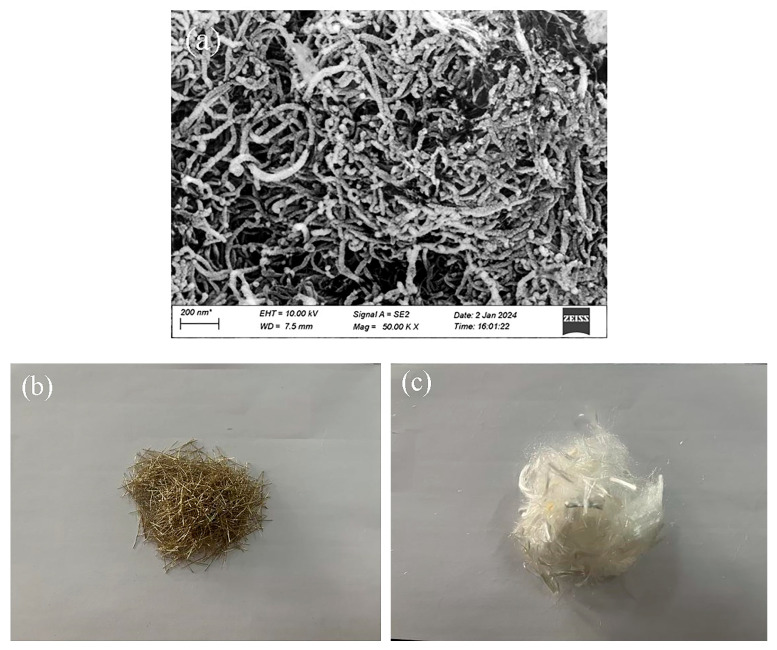
Morphology of hybrid fibers and modified MWCNTs. (**a**) modified MWCNTs, (**b**) Steel fibers, and (**c**) PVA fibers.

The physical composition of the functionalized MWCNTs is analyzed in Table 2, and their appearance is that of black, powdery particles with hydrophilic hydroxyl groups on the surface. FESEM observations revealed that the modified MWCNTs exhibited a curved, entangled, thread-like morphology with a relatively smooth surface, as shown in Figure 3a. The morphological characteristics of PVA fibers and steel fibers are presented in Figure 3b and Figure 3c, respectively. These two types of fibers were incorporated into the test specimens as reinforcement to enhance the system’s mechanical properties. Their physical composition parameters are listed in Table 3; the average lengths of PVA and steel fibers are 12 mm and 13 mm, respectively.

#### 2.1.3. Alkaline Activator Solutions

Sufficient water glass solution (8.98% Na_2_O, 27.2% SiO_2_, and 63.82% H_2_O) was prepared, and an alkaline activator solution was prepared using solid sodium hydroxide (95%) and water glass solution. A mixture of 10 different contents of PVA, steel fibers, and MWCNTs was designed in this study to prepare FL-EGC with FAC and LS for a cement-free system. The raw materials and fiber dosage used to prepare FL-EGC mixtures are shown in Table 4.

Table 4 lists the mass of solid raw materials per cubic meter of composite. The corresponding key mix design parameters, including the sodium silicate modulus (M), alkali equivalent (S), water-to-solid ratio (W), and fly ash replacement rate, are provided in Table 5 for each mixture. The alkaline activator was prepared by mixing solid NaOH (95% purity) with waterglass solution (original modulus 3.24) to achieve the target modulus of 1.4, as calculated using Equations (1) and (2).

#### 2.1.4. Carbon Emissions

The geopolymer produced in this study has a significant reduction in carbon emissions compared to ordinary concrete [49]. The raw materials used are primarily lithium slag, fly ash, silica fume, and other industrial wastes. In the literature on carbon emissions calculations [50], the production of these industrial wastes is assumed to generate no additional carbon emissions. Specifically, for every ton of cement produced, 0.85 to 1 ton of CO_2_ is generated. At the same time, the “carbon footprint” of geopolymers reduces CO_2_ emissions during the production of geopolymer concretes by 50–80% and energy consumption by about 60% compared to concrete made from traditional Portland cement [51]. Therefore, in this study, geopolymers are designed to achieve a green, energy-efficient approach to explore their mechanical properties better.

### 2.2. Preparation of FL-EGC

**Figure 4:**
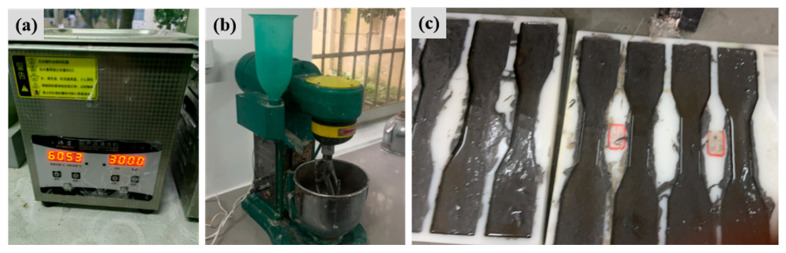
The preparation field process diagram shows (**a**) ultrasonic cleaning, (**b**) cement mortar mixer, and (**c**) preparation of FL-EGC specimen.

The FL-EGC mixtures were prepared according to the proportions in Table 4 and Table 5. For all mixtures, the alkaline activator was prepared with constant parameters: a silicate modulus (M) of 1.4, an alkali equivalent (S) of 7%, and a water-to-solid ratio (W) of 0.4. First, the alkaline activator solution was prepared and allowed to cool; thereafter, the functionalized MWCNTs were dispersed in water using SDS, according to Equations (1) and (2) solid sodium hydroxide of 95% purity was added to the measured water-glass solution (original fraction 3.24) to reduce the modulus of the water-glass solution to 1.4, which is the desired alkaline activator solution. Functionalized MWCNTs and SDS (MWCNTs/SDS = 2:1) were added to 50% of the water required to prepare the specimen. The solution was stirred for 3–9 min at 450 r/min using a mechanical stirrer, after which it was treated with an ultrasonic cleaner at 60 °C for 30 min to disperse the MWCNTs sufficiently, as shown in Figure 4a, which illustrates the operation of the ultrasonic cleaning device.

(1) $$Na_{2}SiO_{3}+2NaOH\to Na_{2}O\cdot SiO_{2}+Na_{2}O+H_{2}O$$

(2) M=1.03×A/D

where A is the percentage of SiO_2_, D is the percentage of Na_2_O, and the molecular weight ratio of SiO_2_ to Na_2_O is 1.032.

Based on the parameters specified in Table 4 and Table 5, the detailed composition of the reference mixture FL-EGC-U (per cubic meter of fresh composite) is provided here. The solid constituents (Table 4) totaled 1700 kg/m^3^ (FA: 1000, MK: 75, SF: 75, LS: 300, FAC: 250). With a fixed water-to-solid ratio W = 0.4 (Table 5), the total water mass was 680 kg. The required alkali equivalent, S = 7%, required 119 kg of Na_2_O, supplied from 125.3 kg of 95% NaOH. Combined with 28 kg of PVA fibers (2 vol.%, density 1.4 g/cm^3^), the estimated fresh density was approximately 2533 kg/m^3^, consistent with a fiber-reinforced mortar. The measured hardened density was below 1950 kg/m^3^, confirming the lightweight design. All other mixtures maintained this base composition while varying the contents of MWCNTs and steel fibers, as shown in Table 4 and Table 5.

Next, as shown in Figure 4b, the cement mortar mixer used in the test was employed to mix the ingredients and produce the geopolymer slurry thoroughly. Add the required raw materials to the cement mortar mixer, mix at low speed for 3 min, then mix in the prepared cooling alkaline solution, the dispersed functionalized MWCNTs solution, and the remaining 50% of water, and continue mixing at high speed for 3 min. Subsequently, PVA and steel fibers were gradually added and mixed at high speed for an additional 6 min to ensure uniform dispersion; mixing was then stopped to form the final geopolymer slurry. Finally, FL-EGC mixture specimens were prepared and cured for molding. The prepared slurry was poured into the molds and vibrated to reduce air content and prevent air bubbles, as shown in Figure 4c for a geopolymer specimen that had been poured but not yet cured. The molds were removed after 24 h of pouring and placed in a curing box (20 ± 2 °C, 95% humidity) for 28 d of curing.

### 2.3. Experimental Design Scheme

Functionalized MWCNTs and hybrid fibers were the two main factors in this experiment, and ten different FL-EGC blend specimens were prepared. The details of the geopolymer specimens are shown in Table 5. Using three different contents of hydroxylated MWCNTs (0.10%, 0.15%, and 0.20% by weight of solids), two distinct contents of PVA (2%, 2.5% by volume of solids), and three different contents of steel fibers (0.5%, 1%, and 1.5% by volume of solids), the addition of dispersants resulted in a better dispersion of the MWCNTs in the alkaline activator solution, and its Bridging and nucleation effects can make the lightweight geopolymer have better compressive and flexural properties, in addition, the bridging effect can better improve the ductility of the polymer, and the modified MWCNTs together with the hybrid fibers can significantly improve its tensile strength. The uniaxial tensile test program is shown in Table 6, and each test block sample will be measured three times in parallel to reduce error and improve test repeatability.

### 2.4. Test Methods

#### 2.4.1. Testing FL-EGC Flowability

**Figure 5:**
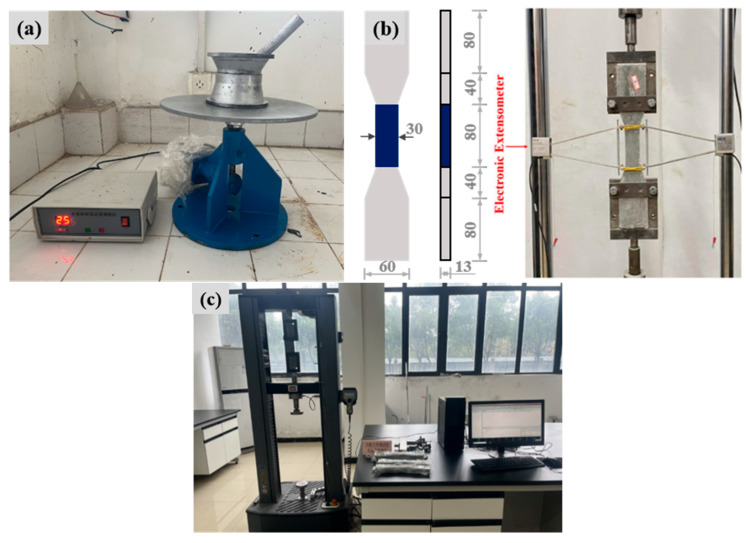
Image of (a) flowability test, (b) geometry and dimensions of the tensile specimen (units: mm), and (c) tension test.

The flowability of FL-EGC without coarse aggregate was measured according to the cement flowability method proposed by the China Building Materials Industry Association [52]. First, the prepared slurry was poured into the test mold in two layers and compacted by vibration using a tamper bar. Tests were performed using a flow meter model NLD-3, as shown in Figure 5a. The table was vibrated 25 times at a frequency of once per second, and the timing was verified with a stopwatch at the same time. At this time, the slurry in the center gradually spread outward under vibration. Finally, immediately after the end of the vibration, the maximum flow diameter of the two directions perpendicular to each other at the bottom of the slurry was measured with a vernier caliper, and the average value was taken to obtain the flowability. Note that this process should be completed within 6 min. The mixture’s flow effect is positively related to the flow diameter. The larger the diameter, the better the slurry flow performance.

#### 2.4.2. Uniaxial Tensile Testing Program

The tensile properties of the geopolymer FL-EGC were assessed using the method proposed in the literature [53]. Figure 5b shows the geometry and dimensions of the dog-bone-shaped tensile specimen used in this study. As shown in Figure 5c, during the field preparation stage of the universal testing machine stretching, the CMT 6103 electronic universal testing machine was used to perform uniaxial tensile tests on the specimens, with a stretching rate of 0.1 mm/min. At the same time, verify that the equipment and the sensor are functioning normally. The sensor will be mounted in the middle section of the test block on both sides. At the beginning of the loading test, the sensor will measure the elongation of the tensile region of the test block, which will be transmitted to the computer for data storage. At the end of the test, the number of cracks ***N***_c_, the average crack width ***W***_c_, and the average crack spacing ***S***_c_ of the tensile specimen were calculated by the crack observer. The calculations were based on Equations (3) and (4)

(3) $$S_{c}=l_{0}/N_{c}$$

(4) $$W_{c}=l/N_{c}$$

where the initial length of the specimen ***l***_0_ is 80 mm, and ***l*** is the total length of the middle section of the specimen at the end of the test in mm.

To monitor crack development in situ, a digital image acquisition system was employed. A high-resolution digital camera equipped with a macro lens was mounted perpendicular to the specimen surface at the gauge section. Images were captured continuously throughout the test at fixed intervals (approximately 1 s), and the image timestamps were synchronized with the mechanical data recorded by the testing machine and the extensometer. This setup enabled the direct correlation between the stress–strain response and the visual evolution of cracking.

#### 2.4.3. FESEM Analysis

FESEM, an electron microscope used to observe microstructural features, was employed to examine the microstructure of the FL-EGC feedstock and reaction products and to analyze them in the context of the experimental process. Since each raw material is a powder, a small amount was taken, encapsulated in a test tube, dispersed in alcohol, and observed after 30 min of standing. After the tensile test, to ensure precise observation, all samples are dried and then gold-coated, and the sample size is determined in accordance with the instrument’s regulations.

## 3. Results and Discussion

### 3.1. FL-EGC Slurry Flowability

**Figure 6:**
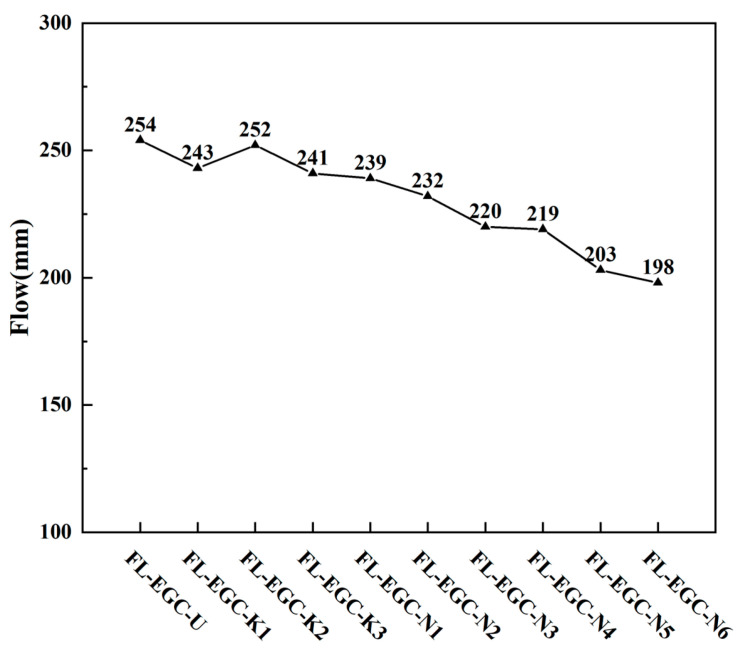
Slurry flow measurements of fiber blends with MWCNTs.

The effect of modified MWCNTs and hybrid fibers on the flowability of the geopolymer slurry is shown in Figure 6. The flow table spread of 254 mm, designated FL-EGC-U, is used as a reference for comparison. The flowability of the geopolymer slurry initially increased and then decreased with increasing functionalized MWCNT content (from 0% to 0.2%). When the MWCNTs content increased to 0.2%, the flowability of FL-EGC-K3 decreased by 5.1%. Therefore, the increase in the content of functionalized MWCNTs adversely affects the flowability of the geopolymer slurry.

The average fluid diameter of the geopolymer slurry decreased as the contents of polyvinyl alcohol and steel fibers increased. The best reinforcement of the mixture specimen (FL-EGC-K2) was achieved when the content of MWCNTs was 0.15%, and that of PVA fibers was 2%. With the increase in steel fiber content, the fluidity of the fresh geopolymer slurry deteriorated, and the FL-EGC-N3 fluidity decreased by 13.4% year-on-year, which was due to the further increase in the specific surface area of the fibers, the adsorption solution capacity became larger, and the adsorption force between the fibers and the matrix became larger. When the PVA fiber content is 2.5%, increasing the steel fiber content worsens slurry flow performance; the FL-EGC-N6 flow is 198 mm, which has the worst flow performance. This is because the high volume fraction of mixed fibers increases the solution’s shear-flow resistance, thereby reducing the fluidity of the geopolymer slurry [54]. The combination of hook-end steel fibers and PVA fibers of similar length increased the shear force with the matrix, and the cohesiveness of the fibers further reduced the flowability but still met the engineering requirements.

### 3.2. Tensile Behavior Analysis of FL-EGC with MWCNTs and PVA Fibers

**Figure 7:**
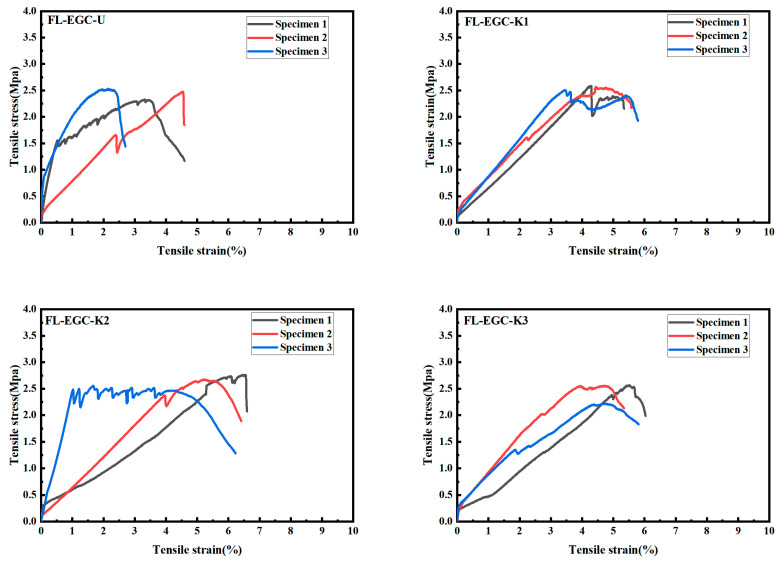
Tensile behavior curve of FL-EGC without steel fiber.

The tensile properties of FL-EGC-U, FL-EGC-K1, FL-EGC-K2, and FL-EGC-K3 were evaluated using uniaxial tensile testing. After curing for 28 days, the test blocks were tested on a universal testing machine. As shown in Figure 7, the tensile data for concrete specimen blocks with modified MWCNTs and PVA are presented. The mixed specimens exhibited pseudo-strain-hardening characteristics similar to those of ECC. The similar stress–strain curves of the FL-ECC mixtures obtained from tests with three parallel samples for specimens with different fiber contents ensured the reliability of the results. The tensile data of the test blocks in this experiment are represented in a trilinear table. In the trilinear module, the initial cracking stress (***σ*_tc_**), initial cracking strain (***ε*_tc_**), ultimate stress (***σ*_tu_**), ultimate strain (***ε*_tu_**), and strain-specific fracture energy (***g*_se_**) are shown. The tensile test was conducted at a loading rate of 0.1 mm/min, and after tensile damage, the specimen failed with a large crack or fracture.

With the increase in the mass fraction of modified MWCNTs, as shown in Table 7, the ultimate stress and strain of the specimens first increased and then decreased. The ultimate stress and ultimate strain of FL-EGC-K2 were the largest, at 2.75 MPa and 5.82%, respectively, and about 11.3% and 40.9% higher than those of FL-EGC-U. As nanoscale materials, carbon nanotubes exhibit excellent nanonucleation and bridging effects, and MWCNTs are fully embedded in the composite matrix, which gradually degrades with increasing loading. As a result, the MWCNTs begin to play a role in connecting the fragile parts and effectively controlling the expansion of microcracks. The nucleation of MWCNTs promotes the formation of geopolymer products, which are more conducive than the bonding of the PVA fiber to the matrix. However, the gradual increase in MWCNTs makes some MWCNTs not sufficiently dispersed, forming agglomerates. Therefore, the addition of modified MWCNTs can significantly increase the tensile strength of the material. Due to the low loading rate, the process of stretching is slow, and the strain-specific fracture energy ***g*_se_** is the amount of energy absorbed by the material during loading, and its value is the integral value of the curve from 0 to ***ε_tu_***, which provides a reference to analyze the mechanical properties of the material. In Table 7, the strain-specific fracture energy of FL-EGC-K2 is twice that of FL-EGC-U. However, all the composites eventually exhibit strain hardening, which is independent of the MWCNT’s content.

**Figure 8:**
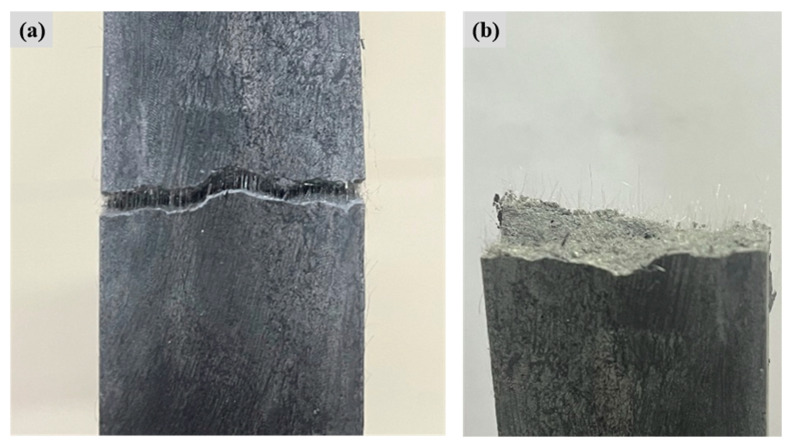
Localized view of FL-EGC-K2 stretching. (**a**) The behavior of PVA fibers before stretching, (**b**) observation of cracks after stretching.

In addition, observations of cracks in tensile specimens were collected to quantify the number of cracks ***N*_c_**, the average crack width ***W*_c_**, and the average crack spacing ***S*_c_**, as shown in Table 8. The crack number ***N*_c_** of the tensile specimens increased and then decreased, with FL-EGC-K2 (59 ± 11) having the highest number of cracks, 2.6 times higher than FL-EGC-U (23 ± 6). The number of cracks increases with specimen ductility, and this increase can warn of and help prevent catastrophic structural collapse when disaster strikes. The crack width of the tensile specimens, 29–34 μm, is relatively well controlled. Compared with references [7,55], ***W*_c_** is about 1/6 of that of the high strain-hardening lightweight ECC (128–180 μm) and about 1/5 of that of the super ductile lightweight EGC (131–147 μm). The crack widths ranged from 0.9 to 2.7 mm. This is mainly because PVA fibers reinforced with functionalized MWCNTs may exhibit stronger fiber-matrix bonding. As shown in Figure 8a, the fibers were connected to the specimen matrix in a bridging manner before pulling off. In Figure 8b, numerous PVA fibers broke and pulled out, exposing them on the surface of the matrix, which was the main reason for the high strain hardening. The results show that, with increasing MWCNT content, both elongation and crack number first increase and then decrease, and the best performance in tensile strength and crack control is FL-EGC-K2, with an MWCNT content of 0.15%.

**Figure 9:**
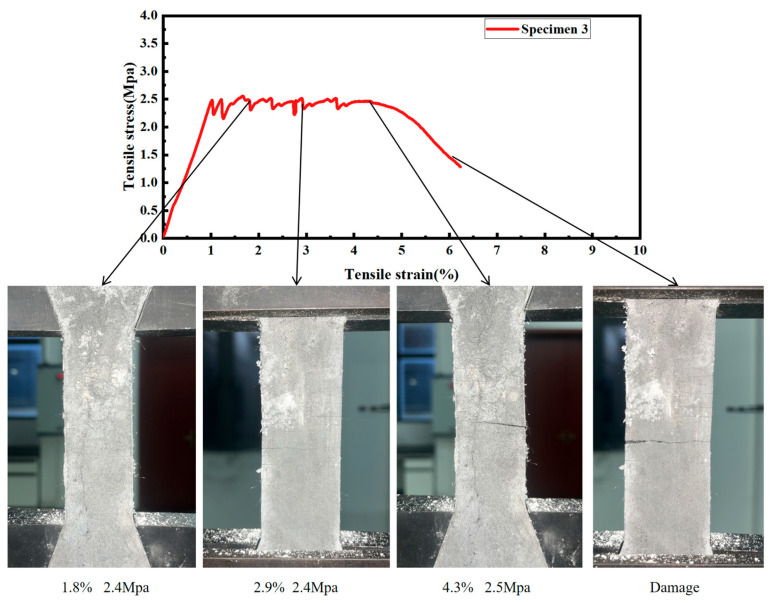
Schematic diagram of crack development during stretching of FL-EGC-K2.

The correlation between the tensile mechanical response and crack evolution is summarized in Figure 9, based on the in situ monitoring described in Section 2.4.2. Representative images corresponding to three characteristic loading stages were selected from the recorded sequence and aligned with their respective points on the stress–strain curve: the end of the linear-elastic (uncracked) stage, the strain-hardening stage characterized by distributed microcracking, and the final failure stage dominated by a localized macrocrack. This visualization directly links the macroscopic constitutive behavior to the microscale damage progression. Accordingly, Figure 9 schematically illustrates that during the strain-hardening stage, increasing strain is accompanied by the formation of multiple fine cracks. At the same time, stress remains relatively stable until final failure occurs via the widening and localization of a significant crack.

The tensile behavior of the FL-EGC mixture specimen can be divided into the no-crack stage, crack-development stage, and crack-extension stage.

(1) During the no-crack stage period, the matrix in the specimen and the PVA fibers, MWCNTs share the force, the matrix of the specimen material bears most of the load at this stage, and the deformation of the specimen follows Hooke’s law until the first crack appears.
(2) Crack development stage period, as the tensile load continues, the stress increases slowly or remains unchanged, the strain gradually increases, and new cracks are continuously generated. PVA fibers and modified MWCNTs bridge the broken matrix to withstand the load.
(3) During the crack expansion period, the specimen matrix suffers damage. It loses its load-bearing capacity, and a large number of PVA fibers and MWCNTs are pulled out and fractured. The specimen cracks gradually become more pronounced until the tensile specimen fails under load.

To further demonstrate the effectiveness of the proposed FL-EGC composites, their performance was compared with values reported in the literature. Conventional ECCs typically exhibit tensile strains in the range of 2–5% with crack widths between 80 and 150 μm [7,55], while lightweight EGCs often reach tensile strains of 1–3% [21,22,29]. In contrast, the optimized FL-EGC mixture in this study (2.5% PVA + 1.0% steel fibers + 0.15% MWCNTs) achieved a tensile strain of 3.02% and maintained average crack widths below 60 μm, indicating superior crack control. Moreover, the strain-specific fracture energy of FL-EGC (91.21 KJ/m^3^) was significantly higher than that of reported fiber-reinforced geopolymers without nanomaterials [49,54]. These comparisons confirm that the synergy between functionalized MWCNTs and hybrid fibers not only improves the tensile properties of FL-EGC but also places its performance on par with, or better than, existing engineered geopolymer composites.

### 3.3. Tensile Behavior Analysis of PVA Fiber FL-EGC with MWCNTs and Steel

**Figure 10:**
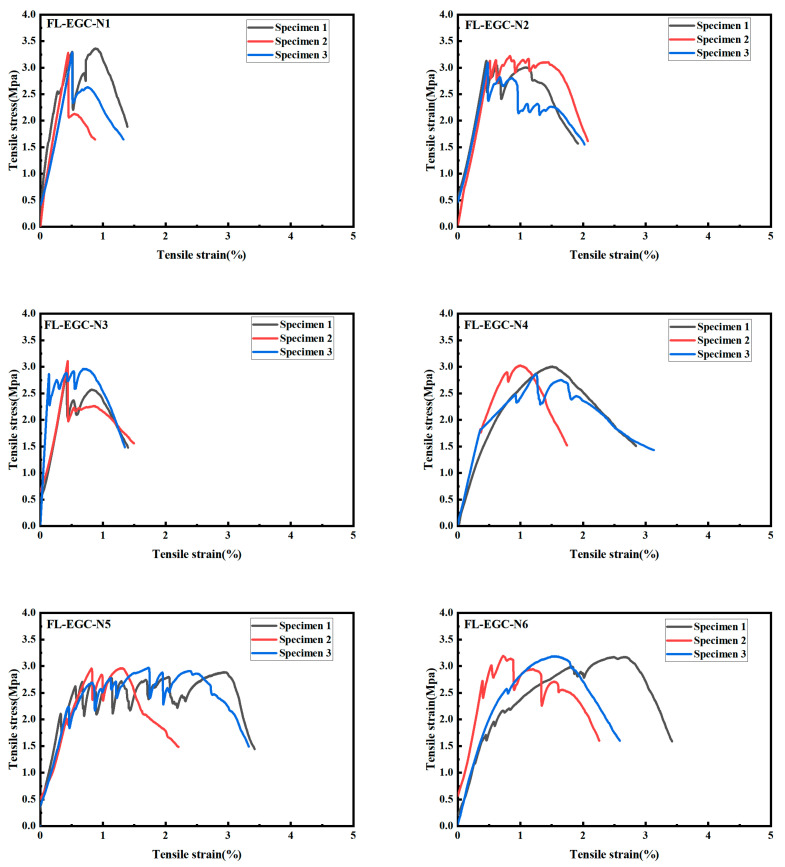
FL-EGC tensile data curve with steel material.

In Section 3.2, the results of uniaxial tensile tests showed that MWCNTs with a mass fraction of 0.15% exhibited the best stress–strain capacity. Therefore, in this section, the effect of varying hybrid fiber content on the tensile behavior of FL-EG blends is analyzed. In Table 4, the blending ratios of raw fibers for all samples are presented. The tensile test results for FL-EGC specimens with hybrid fibers are shown in Figure 10.

A comparison across six different fiber ratios indicates that, for a given steel fiber content, the fluctuation of the tensile curves decreases, approximately inversely proportional to the steel fiber content. This suggests that the addition of steel fibers can effectively reduce stress fluctuations during tensile testing. With the addition of steel and polyvinyl alcohol fibers, tensile strength and ductility can be increased. Meanwhile, these geopolymer tensile specimens exhibit cracking behavior and strain hardening consistent with those of the steel-added tensile specimens from the crack-free stage through failure, compared with the tensile specimens without steel addition.

Table 9 analyzes the tensile properties of the specimens with the same MWCNTs (0.15%) and different contents of hybrid fibers. When MWCNTs are 0.15%, and PVA content is 2%, the ultimate stress of geopolymer decreases with the increase in steel fiber content, and the ultimate strain increases and then decreases. When the PVA content is 2.5%, with the rise in steel fiber content, the ultimate stress of geopolymer specimens gradually increases, and the ultimate strain follows the same trend as the value of the strain-specific fracture energy; both increase first and then decrease, and the strain-specific fracture energy of FL-EGC-N5 is the maximum value of 91.21 KJ/m^3^. The FL-EGC-H5 data indicate that a geopolymer material containing 0.15% MWCNTs and 1.0% steel fibers exhibited the highest strain energy.

Compared with specimens without steel fibers, the tensile strength of FL-EGC with steel and PVA fibers increased, whereas specimen deformability decreased. This may be related to the properties of the hybrid fibers; on the one hand, PVA shows excellent deformation ability, and its low elastic modulus effectively inhibits the development of fine cracks; on the other hand, due to the high elastic modulus of the steel fibers, it provides the specimens with high mechanical strength, but the deformation ability is relatively poor. The results show that with the addition of hybrid fibers, the ultimate stress and ultimate strain values of the tensile materials containing hybrid fibers have a relatively significant increase, which is significantly higher than those of the tensile materials containing only PVA fibers, but the deformation capacity decreases. The optimal steel fiber content was 1.0% under the specified PVA volume fraction.

As shown in Table 10, the quantified crack data were obtained by calculating the crack length after observation using a magnified viewer. With increasing steel fiber content, the number of hybrid fiber FL-EGC cracks first increases from 13 to 35, then decreases to 18. Compared with the results of FL-EGC tensile specimens containing only PVA, the number of hybrid fiber FL-EGC cracks decreases. The number of FL-EGC-N5 cracks is the highest, at about 1/2 of that of FL-EGC-K2. The average crack width of the hybrid fiber FL-EGC is in the range of 49~68 μm, which still shows reasonable crack control. The range of hybrid fiber FL-EGC was 1.06~3.16 mm. In conclusion, hybrid fiber FL-EGC exhibited lower ductility than FL-EGC containing only PVA but retained superior ductility and crack-control capability. The highest number of cracks was observed in FL-EGC-N5, which included 1.5% steel fibers.

### 3.4. Microstructure of FL-EGC Blends

#### 3.4.1. Microstructure of FL-EGC with MWCNTs and PVA Fibers

The microstructural characteristics of FL-EGC incorporating MWCNTs and PVA fibers were investigated using FESEM in order to qualitatively examine fiber–matrix interaction and interfacial features after uniaxial tensile testing. Compared with specimens without fiber addition, the incorporation of PVA fibers and MWCNTs was associated with improved tensile response and crack control behavior at the macroscopic level. The following discussion is therefore limited to qualitative microstructural observations.

**Figure 11:**
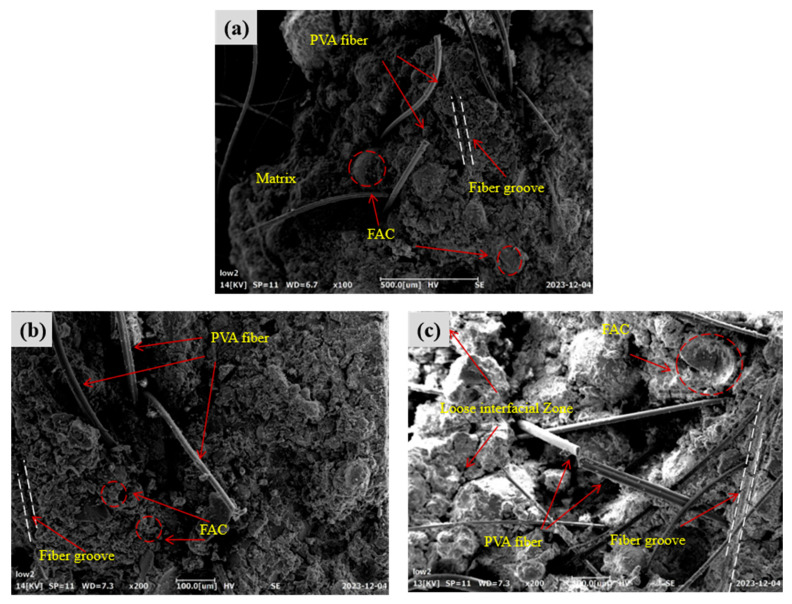
Morphological characteristics of PVA fibers in specimen FL-EGC-K2; (**a**) PVA fiber pull-out and elongation behavior; (**b**) Matrix grooves caused by fiber–matrix friction; (**c**) Fiber–matrix interfacial damage and slip-hardening features.

As shown in Figure 11a, FESEM images of specimen FL-EGC-K2 reveal PVA fibers partially embedded within the matrix, with one end anchored in the matrix and the other exhibiting elongation after tensile loading. Groove-like traces can be observed on the matrix surface adjacent to the fibers (Figure 11b,c), which are attributed to frictional interaction between the fibers and the surrounding matrix during the pull-out process. Such features are commonly associated with progressive fiber slip accompanied by increasing pull-out resistance, a phenomenon often referred to as slip hardening [56].

With continued tensile loading, fiber bending and deformation occur, which may delay abrupt fiber pull-out. When the applied load exceeds the load-carrying capacity of the fibers, fiber rupture is observed. As shown in Figure 11, fibers remain embedded within the matrix after cracking, while localized interfacial damage and matrix fragmentation can be identified. At the damaged interfaces, fiber bridging across cracks is observed, indicating that load transfer between the fibers and the matrix can still occur after matrix cracking.

Residual matrix material adhering to the fiber surface suggests the presence of interfacial bonding between PVA fibers and the geopolymer matrix. These observations imply that PVA fibers contribute to deformation accommodation and crack-bridging behavior; however, it should be noted that the relatively low elastic modulus of PVA fibers may limit their contribution to tensile strength enhancement.

#### 3.4.2. Microstructure of FL-EGC with MWCNTs and Hybridized Fibers

**Figure 12:**
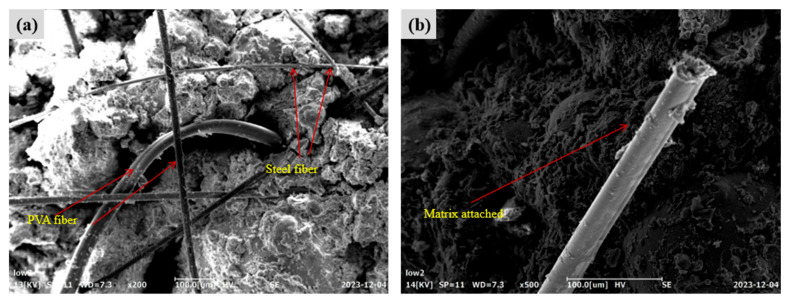
Morphological characteristics of steel fibers in specimen FL-EGC-N5; (**a**) Embedded hybrid fibers and interfacial morphology; (**b**) Matrix adhesion and anchorage of hooked-end steel fibers.

The microstructure of FL-EGC incorporating hybrid fibers in combination with MWCNTs was further examined to qualitatively assess interfacial morphology and crack-bridging characteristics. Representative FESEM images of specimen FL-EGC-N5 are shown in Figure 12. As illustrated in Figure 12a, both PVA fibers and steel fibers are embedded within the matrix, while localized regions with relatively loose interfacial features can be observed. Such regions may be susceptible to matrix fragmentation under increasing external load. In Figure 12b, residual matrix material adhering to the surface of steel fibers is evident, suggesting effective mechanical interlocking and interfacial bonding. In addition, the hooked-end geometry of the steel fibers provides anchorage, which is expected to contribute to enhanced load transfer during tensile loading.

**Figure 13:**
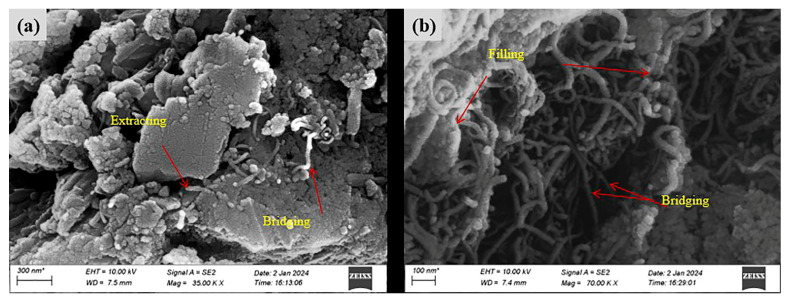
Morphological features of functionalized MWCNTs in specimen FL-EGC-N5; (**a**) MWCNT pull-out and crack-bridging features; (**b**) Microcrack bridging by clustered MWCNTs.

Figure 13 presents FESEM observations of MWCNTs in the matrix of specimen FL-EGC-N5 after tensile damage. As shown in Figure 13a, functionalized MWCNTs exhibit pull-out and crack-bridging features at the microscale. In some regions, MWCNTs are observed within the matrix adjacent to crack surfaces, which may hinder crack opening and propagation locally. In Figure 13b, clusters of MWCNTs can be identified bridging microcracks, with both ends embedded in the matrix, indicating their participation in load transfer at the microscale.

It should be emphasized that these observations provide qualitative evidence of MWCNT participation in crack-bridging behavior, rather than a quantitative assessment of dispersion quality. While the presence of MWCNT pull-out and bridging features suggests a potential contribution to crack resistance, localized aggregation of MWCNTs is also observed, and its influence on macroscopic performance requires cautious interpretation. Therefore, the role of functionalized MWCNTs in FL-EGC is discussed here in terms of microstructural characteristics and possible implications, rather than definitive enhancement mechanisms.

### 3.5. Interfacial Mechanism of Hybrid Fibers Co-Modified with MWCNTs to Enhance FL-EGC

**Figure 14:**
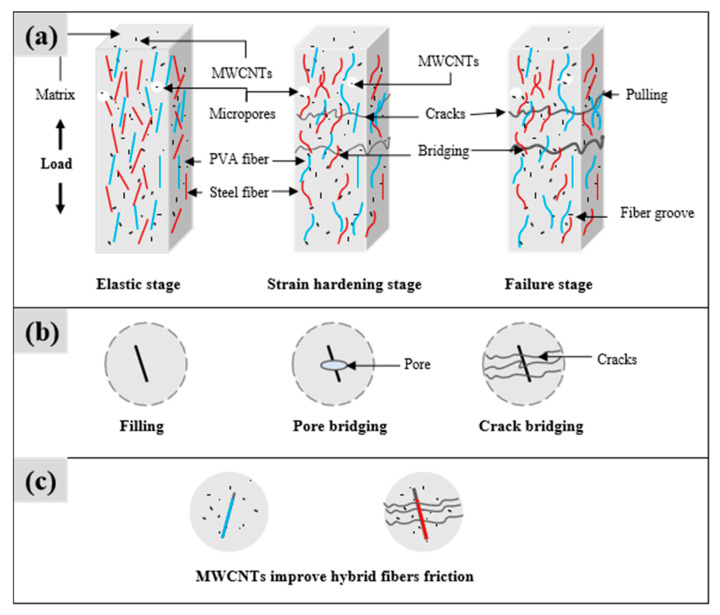
An interfacial pattern of FL-EGC enhanced by hybrid fibers synergized with MWCNTs; (**a**) Stage-dependent tensile interfacial behavior of FL-EGC (schematic); (**b**) MWCNTs near microcracks and pores after tensile damage; (**c**) MWCNT-related fiber–matrix interfacial interactions.

Based on the results of the uniaxial tensile tests and the qualitative microstructural observations, the interfacial behavior of FL-EGC incorporating hybrid fibers and MWCNTs is discussed from a microscopic perspective, as schematically illustrated in Figure 14. It should be emphasized that the following analysis represents an interpretative framework derived from experimental observations, rather than a definitive mechanistic model.

Under quasi-static tensile loading, the tensile response can be generally divided into three stages: elastic stage, strain-hardening stage, and failure stage. As shown in Figure 14a, during the elastic stage, both the geopolymer matrix and the embedded fibers participate in load bearing, with the matrix carrying the primary load before visible cracking occurs. The deformation behavior in this stage approximately follows linear elastic characteristics.

As tensile loading progresses into the strain-hardening stage, multiple cracks initiate and gradually propagate. Hybrid fibers and MWCNTs begin to participate more actively in load transfer across cracked sections. At this stage, frictional interactions may occur between fibers and the surrounding matrix, as well as between MWCNTs and the matrix. These interactions are inferred from the observed pull-out traces and interfacial features shown in Figure 11 and Figure 12. In particular, the hydrophilic nature of PVA fibers and their surface morphology may contribute to enhanced interfacial friction, while steel fibers provide mechanical anchorage through their hooked ends. These features are consistent with the observed crack-bridging behavior but are discussed here as qualitative observations rather than quantified mechanisms.

With continued loading, crack widths increase, and partial pull-out or rupture of PVA fibers and MWCNTs can be observed. At higher load levels, steel fibers increasingly contribute to load transfer due to their anchorage effect and frictional resistance. In the final failure stage, extensive fiber pull-out and straightening of steel fibers occur until macroscopic failure is reached. Damaged PVA and steel fibers observed on the fracture surface (Figure 8) further support this interpretation.

Figure 14b presents representative FESEM images of MWCNTs in the tensile-damaged matrix, illustrating their presence near microcracks and pores. These observations suggest that MWCNTs may participate in crack-bridging and defect interaction at the microscale. However, considering that pores and microcracks may also originate from air entrapment during specimen preparation and from localized damage during tensile loading, the role of MWCNTs is discussed here in terms of potential microstructural interaction, rather than confirmed defect elimination.

As illustrated in Figure 14c, MWCNTs are observed in association with fiber–matrix interfaces, forming additional microscale interaction zones. These interfaces may contribute to energy dissipation through friction and interlocking during tensile deformation. Nevertheless, due to the absence of quantitative dispersion characterization and direct interfacial strength measurements, such effects are interpreted cautiously and limited to qualitative implications within the scope of the present study.

Overall, the interfacial analysis indicates that the combined presence of hybrid fibers and MWCNTs is associated with complex multi-scale interactions at the microscopic level, which may contribute to the observed tensile behavior. Definitive conclusions regarding reinforcement mechanisms require further experimental validation.

## 4. Conclusions

This study investigated the influence of functionalized MWCNTs in combination with PVA and steel fibers on the tensile behavior, crack characteristics, and microstructural features of lithium slag–based geopolymer composites (FL-EGC). Within the experimental scope of this work, the following conclusions can be drawn:

1. The incorporation of functionalized MWCNTs and hybrid fibers reduced the flowability of FL-EGC due to their high specific surface area and interaction with the alkaline solution; however, the mixtures remained workable and suitable for laboratory-scale casting.
2. Within the tested range, the addition of functionalized MWCNTs at an optimal dosage of 0.15% was associated with increases in ultimate tensile stress, ultimate tensile strain, and strain-specific fracture energy. These improvements are discussed in relation to observed microstructural features, such as crack-bridging and interfacial interaction, rather than as direct mechanistic proof.
3. The combined use of PVA fibers, steel fibers, and MWCNTs resulted in improved tensile performance compared with single-fiber systems. PVA fibers primarily contributed to crack control and deformation capacity, while steel fibers enhanced load-bearing capacity through mechanical anchorage. Excessive steel fiber content was observed to reduce deformability.
4. Crack spacing and crack width were effectively controlled in the hybrid fiber–MWCNT system, with finer crack distribution observed compared to conventional ECC/EGC materials. These results indicate enhanced crack management behavior under tensile loading.
5. FESEM observations revealed fiber pull-out, crack-bridging features, and matrix adhesion at fiber surfaces, as well as the presence of MWCNTs near microcracks and pores. These observations provide qualitative microstructural evidence supporting the tensile behavior trends, but do not constitute quantitative validation of reinforcement mechanisms.

In summary, this study demonstrates that the combined incorporation of functionalized MWCNTs and hybrid fibers can improve the tensile response and crack control behavior of lithium slag–based geopolymers within the investigated parameter range. The conclusions are intentionally limited to tensile performance and qualitative microstructural interpretation. Future work will focus on systematic evaluation of compressive and durability properties, quantitative dispersion characterization, and micromechanical modeling to further clarify reinforcement mechanisms and assess structural applicability.

## Figures and Tables

**Table 1:** Chemical composition of cementitious materials and LS (wt%).

Chemical Composition	FA	MK	SF	LS	FAC
SiO_2_	45.1	53.00	92.2	25.79	50.6
AI_2_O_3_	24.2	41.50	3.17	16.84	27.1
CaO	6.45	/	0.31	29.17	2.8
MgO	/	0.49	0.11	0.56	1.2
Fe_2_O_3_	0.85	0.80	0.19	2.68	7.1
Na_2_O	1.2	0.18	/	0.28	0.5
K_2_O	/	0.13	/	3.87	1.3
SO_3_	2.1	/	/	9.75	0.3
Li_2_O	/	/	/	0.26	/
Others	2.8	3.9	3.92	10.8	8.2

**Table 2:** Physical properties of modified MWCNTs in this study.

Capability	Diameter (nm)	Inside Diameter (nm)	Lengths (μm)	Density (g/cm^3^)	Specific Surface Area (m^2^/g)
MWCNTs	10–15	5–8	2–8	0.09	≫190

**Table 3:** Physical properties of two different fibers.

Fibroid	Calibre (μm)	Lengths (mm)	Dissociation (MPa)	Modulus (GPa)	Densities (g/cm^3^)
Steel	220	13	3000	214	7.9
PVA	60	12	1720	52.6	1.4

**Table 4:** Composition of composite FL-EGC mixtures (kg/m^3^ for solids; fiber: vol.%; MWCNT: wt.%).

Mixture Specimen	FA	MK	SF	LS	FAC	MWCNTs (%)	PVA(%)	Steel (%)
FL-EGC-U	1000	75	75	300	250	0	2	0
FL-EGC-K1	1000	75	75	300	250	0.10	2	0
FL-EGC-K2	1000	75	75	300	250	0.15	2	0
FL-EGC-K3	1000	75	75	300	250	0.2	2	0
FL-EGC-N1	1000	75	75	300	250	0.15	2	0.5
FL-EGC-N2	1000	75	75	300	250	0.15	2	1
FL-EGC-N3	1000	75	75	300	250	0.15	2	1.5
FL-EGC-N4	1000	75	75	300	250	0.15	2.5	0.5
FL-EGC-N5	1000	75	75	300	250	0.15	2.5	1
FL-EGC-N6	1000	75	75	300	250	0.15	2.5	1.5

**Table 5:** Arrangement of test groups for hybrid modified MWCNTs and hybrid fiber composites.

Mixture Specimen	Sodium Silicate Solution Modulus (M)	Alkali Equivalent (S)	Water-Solid Ratio (W)	Fly Ash Replacement Rate (%)	Modified Multi-Walled Carbon Nanotube Content (%)	PVA Fiber Content (%)	Steel Fiber (%)
FL-EGC-U	1.4	7%	0.4	20	0	2	0
FL-EGC-K1	1.4	7%	0.4	20	0.10	2	0
FL-EGC-K2	1.4	7%	0.4	20	0.15	2	0
FL-EGC-K3	1.4	7%	0.4	20	0.2	2	0
FL-EGC-N1	1.4	7%	0.4	20	0.15	2	0.5
FL-EGC-N2	1.4	7%	0.4	20	0.15	2	1
FL-EGC-N3	1.4	7%	0.4	20	0.15	2	1.5
FL-EGC-N4	1.4	7%	0.4	20	0.15	2.5	0.5
FL-EGC-N5	1.4	7%	0.4	20	0.15	2.5	1
FL-EGC-N6	1.4	7%	0.4	20	0.15	2.5	1.5

**Table 6:** Test program for composite specimen.

Mixture specimen	FL-EGC-U	FL-EGC-K1	FL-EGC-K2	FL-EGC-K3	FL-EGC-N1
Number of parallel tests	3	3	3	3	3
Mixture specimen	FL-EGC-N2	FL-EGC-N3	FL-EGC-N4	FL-EGC-N5	FL-EGC-N6
Number of parallel tests	3	3	3	3	3

**Table 7:** Tensile data for composite specimens without steel.

Mixture Number	*σ*_tc_ (MPa)	*ε*_tc_ (%)	*σ*_tu_ (MPa)	*ε*_tu_ (%)	*g*_se_ (KJ/m^3^)
FL-EGC-U	1.49 ± 0.41	0.51 ± 0.02	2.44 ± 0.01	3.44 ± 0.91	53.91 ± 6.03
FL-EGC-K1	1.67 ± 0.83	2.44 ± 0.03	2.57 ± 0.04	4.39 ± 0.81	62.32 ± 4.25
FL-EGC-K2	1.87 ± 0.91	1.01 ± 0.51	2.75 ± 0.10	5.82 ± 0.71	113.89 ± 8.96
FL-EGC-K3	1.52 ± 0.26	1.98 ± 0.53	2.45 ± 0.16	5.12 ± 0.33	84.51 ± 10.61

**Table 8:** Average cracking parameters of FL-EGC without steel fibers.

Mixture Number	*N*_c_	*W*_c_ (μm)	*S*_c_ (mm)
FL-EGC-U	23 ± 6	34.02 ± 4.61	2.70 ± 0.54
FL-EGC-K1	34 ± 9	31.07 ± 7.32	2.11 ± 0.18
FL-EGC-K2	59 ± 11	29.26 ± 6.71	0.90 ± 0.18
FL-EGC-K3	41 ± 9	32.36 ± 2.67	1.04 ± 0.04

**Table 9:** Tensile data of steel-containing composite specimens.

Mixture Number	*σ*_tc_ (MPa)	*ε*_tc_ (%)	*σ*_tu_ (MPa)	*ε*_tu_ (%)	*g*_se_ (KJ/m^3^)
FL-EGC-N1	2.51 ± 0.39	0.32 ± 0.01	3.36 ± 0.16	1.01 ± 0.99	35.32 ± 5.87
FL-EGC-N2	2.91 ± 0.18	0.42 ± 0.03	3.24 ± 0.31	1.42 ± 0.59	46.43 ± 7.34
FL-EGC-N3	2.78 ± 0.25	0.13 ± 0.01	3.12 ± 0.19	0.89 ± 0.84	29.75 ± 6.03
FL-EGC-N4	2.79 ± 0.47	0.51 ± 0.01	2.96± 0.06	1.67 ± 0.26	59.21 ± 6.84
FL-EGC-N5	2.29 ± 0.09	0.40 ± 0.13	2.99 ± 0.10	3.02 ± 1.02	91.21 ± 9.35
FL-EGC-N6	2.31 ± 0.20	0.34 ± 0.02	3.11 ± 0.18	2.68 ± 1.10	79.47 ± 5.86

**Table 10:** Average cracking parameters of FL-EGC with steel fibers.

Mixture Number	*N*_c_	*W*_c_ (μm)	*S*_c_ (mm)
FL-EGC-N1	13 ± 6	68.43± 12.54	3.16 ± 1.21
FL-EGC-N2	17 ± 4	58.26 ± 4.28	2.68 ± 0.59
FL-EGC-N3	11 ± 6	64.35 ± 9.86	3.04 ± 1.71
FL-EGC-N4	28 ± 5	49.92 ± 6.14	1.06 ± 0.75
FL-EGC-N5	35 ± 11	51.46 ± 7.40	1.30 ± 0.83
FL-EGC-N6	18 ± 9	54.65 ± 3.27	1.58 ± 0.79

## Data Availability

The original contributions presented in this study are included in the article. Further inquiries can be directed to the corresponding author.
